# The Application of Polymeric Nanoparticles as Drug Delivery Carriers to Cells in Neurodegenerative Diseases

**DOI:** 10.1111/cpr.13804

**Published:** 2025-02-11

**Authors:** Lian Jin, Libo Nie, Yan Deng, Ghulam Jilany Khana, Nongyue He

**Affiliations:** ^1^ Hunan Key Laboratory of Biomedical Nanomaterials and Devices Hunan University of Technology Zhuzhou Hunan People's Republic of China; ^2^ Shenzhen Feidu Biomedicine Co. Ltd. Shenzhen China; ^3^ Institute for Future Sciences University of South China Changsha China; ^4^ Institute of Cytology and Genetics, School of Basic Medical Sciences, Hengyang Medical School University of South China Hengyang China; ^5^ Faculty of Pharmaceutical Sciences University of Central Punjab Lahore Pakistan; ^6^ State Key Laboratory of Digital Medical Engineering, School of Biological Science and Medical Engineering Southeast University Nanjing China

**Keywords:** drug delivery, gene delivery, nanoparticles, neurodegenerative diseases, polymer

## Abstract

In spite of great advances in modern medicine, there are a few effective strategies for the treatment of neurodegenerative diseases characterised by neuron loss or degeneration. This results from complex pathogenesis of the diseases and the limited drug uptake of the brain due to the presence of blood‐brain barrier. Nanoparticle‐based drug delivery systems are expected to improve the drug utilisation. Polymeric nanoparticles represent promising drug delivery carriers to the brain due to their unique advantages such as good biodegradability and biocompatibility, flexibility in surface modification and nontoxicity. In addition, the delivery of genetic drugs may stop the progression of neurodegenerative diseases at the genetic level and even avoid the irreversible damage in the central nervous system. In this review, an overview of studies on polymer‐based nanoparticles for drug delivery to the central nervous system in typical neurodegenerative diseases, especially Alzheimer's diseases and Parkinson's diseases, is described. Meanwhile, their applications in gene delivery in these disorders are discussed. And the challenges and future perspectives for the development of polymeric nanoparticles as drug delivery carriers in neurodegenerative diseases are concluded.

## Graphical abstract

Polymeric nanoparticles represent promising drug (gene) delivery carriers to the brain due to their unique advantages to facilitate the penetration of blood–brain barrier and endocytosis of neuronal cells in neurodegenerative diseases.

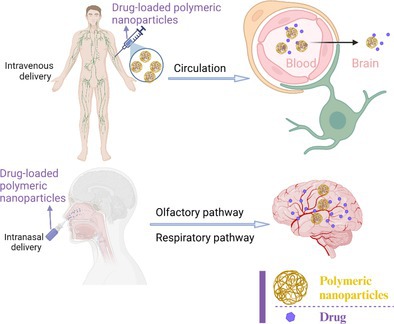

## 1 Introduction

Neurodegenerative diseases are a heterogeneous group of complex diseases characterised by neuronal loss and progressive degeneration of neurons in the central or peripheral nervous system [1, 2]. The most prevalent ones are Alzheimer's disease (AD), Parkinson's disease (PD), Huntington's disease (HD), amyotrophic lateral sclerosis (ALS) and multiple sclerosis (MS) [3, 4, 5]. The central nervous system (CNS) consists of the brain and spinal cord and plays a vital role in the function and regulation of the body [6, 7]. At the cellular level, neurons that harbour extensive cell–cell communication capabilities are basic functional units of the CNS [8] and glia cells including oligodendrocytes, astrocytes and microglia are the most abundant cell type of the CNS [9, 10, 11]. These cells are extra‐sensitive to temperature fluctuations, pathogens and toxins [12]. Notably, their damage is normally irreversible [13].

**FIGURE 1:**
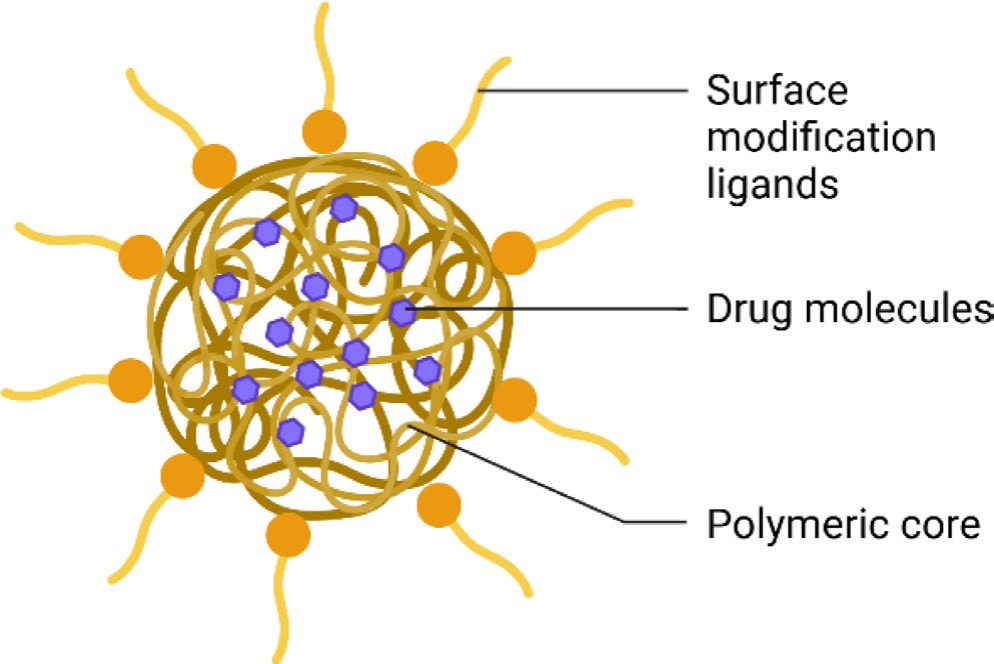
Basic structure of a polymeric nanoparticle.

It is extremely difficult to transport the drugs or chemicals into the CNS because of its natural barriers such as the blood–brain barrier (BBB), the blood–cerebrospinal fluid barrier and the cerebrospinal fluid–blood barrier [14, 15]. The BBB serves as a vital regulatory system that controls the transport of ions, nutrients and drugs between the CNS and the bloodstream [16]. It precludes large molecules and 98% of small molecules from entering the brain [17]. To efficiently transport drugs across the BBB and guarantee the safety of the cells, nanosized drug delivery carriers illustrate great potential [18]. Nanotechnology has been extensively used for a variety of applications including DNA/RNA detection, cancer diagnosis and personalised medication [19, 20, 21]. Particularly, it displays immense potential in addressing the complex needs for the treatment of neurological disorders, such as the penetration of the BBB and consequential drug delivery to cells of interest [22, 23]. A series of nanoparticles have been used as the vectors transporting drugs cross the cell membrane and entering cells [24]. They protect the drug from enzymatic degradation, reduce the systemic clearance, increase the bioavailability in the brain and facilitate drug targeting to a specific region of the brain to reduce the side effects [25]. They can be classified into viral and non‐viral carriers and non‐viral carriers contain cationic lipids and polymers. Viral carriers rely on the innate ability of viruses to reach and penetrate specific cellular targets and express genetic information into host cells [26]. However, their potential risks such as immunogenicity limit their capacity for carrying drugs [27], and the possible damage to cells in the CNS excludes viral carriers from feasible strategies for neurodegenerative diseases. Non‐viral vectors possess obvious advantages such as reduced pathogenicity, low cost and simple production techniques [28, 29]. Among them, polymeric nanoparticles (Figure 1), in particular, are a promising choice as drug delivery platform for CNS targeting due to their remarkable advantages including tunable size, nontoxicity, biocompatibility, biodegradability, controllable drug release and easy modification with specific ligands for improved transcytosis efficiency [30, 31]. Moreover, polymeric nanoparticles have a prolonged half‐life time, which can increase drug uptake by the CNS [32, 33]. In addition, polymeric nanoparticles are versatile to deliver a wide range of drugs such as peptides, nucleic acids and proteins via different bonding approaches including non‐covalent bonding (hydrophobic effects, hydrogen‐bonding and electrostatic interactions) and covalent crosslinking [34, 35, 36, 37]. Collectively, quite a lot of studies have confirmed the unique advantages of polymeric nanoparticles in delivering drugs to the CNS [38, 39]. In this review, we will provide an overview on the application of polymeric nanoparticles as drug delivery carriers in neurodegenerative diseases, especially AD and PD. The polymeric nanoparticle–based strategies for the early intervention will also be summarised, with a focus on gene therapy. In addition, the research challenges and future directions in this field will be concluded.

## 2 Administration Routes of Drugs to the CNS

**FIGURE 2:**
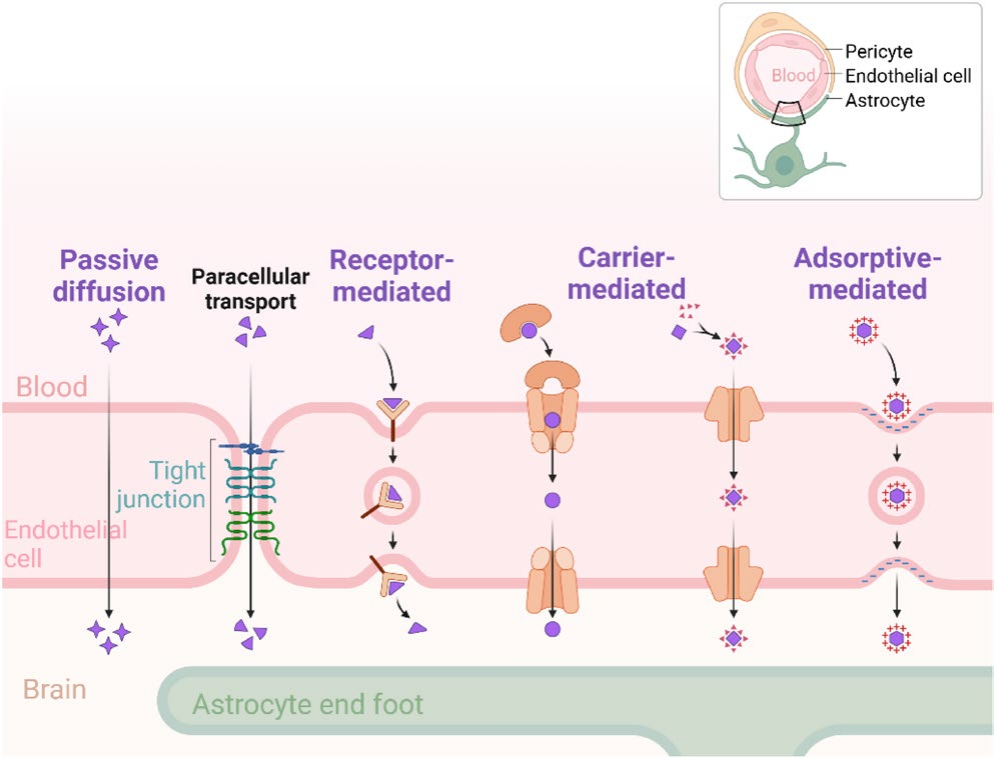
Solute transport mechanisms across BBB.

During the polymeric nanoparticle‐mediated drug delivery to the CNS, the administration route is found tightly related to the delivery efficiency. Presently, a group of direct and indirect routes including oral, intravenous, intracerebroventricular and intranasal administrations have been proposed [40]. When administered orally, the majority of therapeutic agents are rapidly degraded by the first‐pass effect of gastrointestinal and hepatic enzymes [41, 42], which dramatically restricted the application of this route, especially for polymer‐based nanoparticles. When administered intravenously, a proportion of medications are cleared by the immune cells or other components in the blood [43]. The remaining part has to cross the BBB, the highly selective and strict structure. As shown in Figure 2, the transport mechanisms of the BBB include passive diffusion, carrier‐mediated transport, receptor‐mediated transport and adsorptive transcytosis [40]. Passive diffusion is only for lipid‐soluble small molecules under 400 Da [44]. Most substances cross the BBB through carrier‐ and receptor‐mediated transport via their strong affinity to specific carriers or receptors on the surface of BBB [45]. Adsorptive‐mediated transcytosis is realised by electrostatic interactions between the positive charge of substances and negative charge on the plasma membrane of endothelial cells [46, 47]. To improve the stability, the escape ability from immune system and the BBB crossing capability of intravenously administrated nanoparticles in neurodegenerative diseases, various types of modifications have been exploited including stabilisers, enzymatic inhibitors and BBB penetration promoters [48, 49, 50]. Polyethyleneglycol (PEG) is the most widely used modification ligand to date as it stabilises nanoparticles in biological media such as blood [51]. It is also recognised that a PEG coating protects nanoparticles from the attack of the immune system such as the macrophages, eventually avoiding circulation clearance [52]. Macrophages and T cells have also been utilised to help polymeric nanoparticles escape from clearance by the immune system [48]. A range of ligands (e.g., peptides, proteins, aptamers, small molecules and antibodies) have been linked to polymeric nanoparticles to target specific cell types such as neurons, astrocytes and endothelial cells of the BBB [53]. They can increase the drug bypass of the BBB by receptor‐mediated endocytosis. Nevertheless, current research has not achieved satisfying results, which means more efforts are required.

**FIGURE 3:**
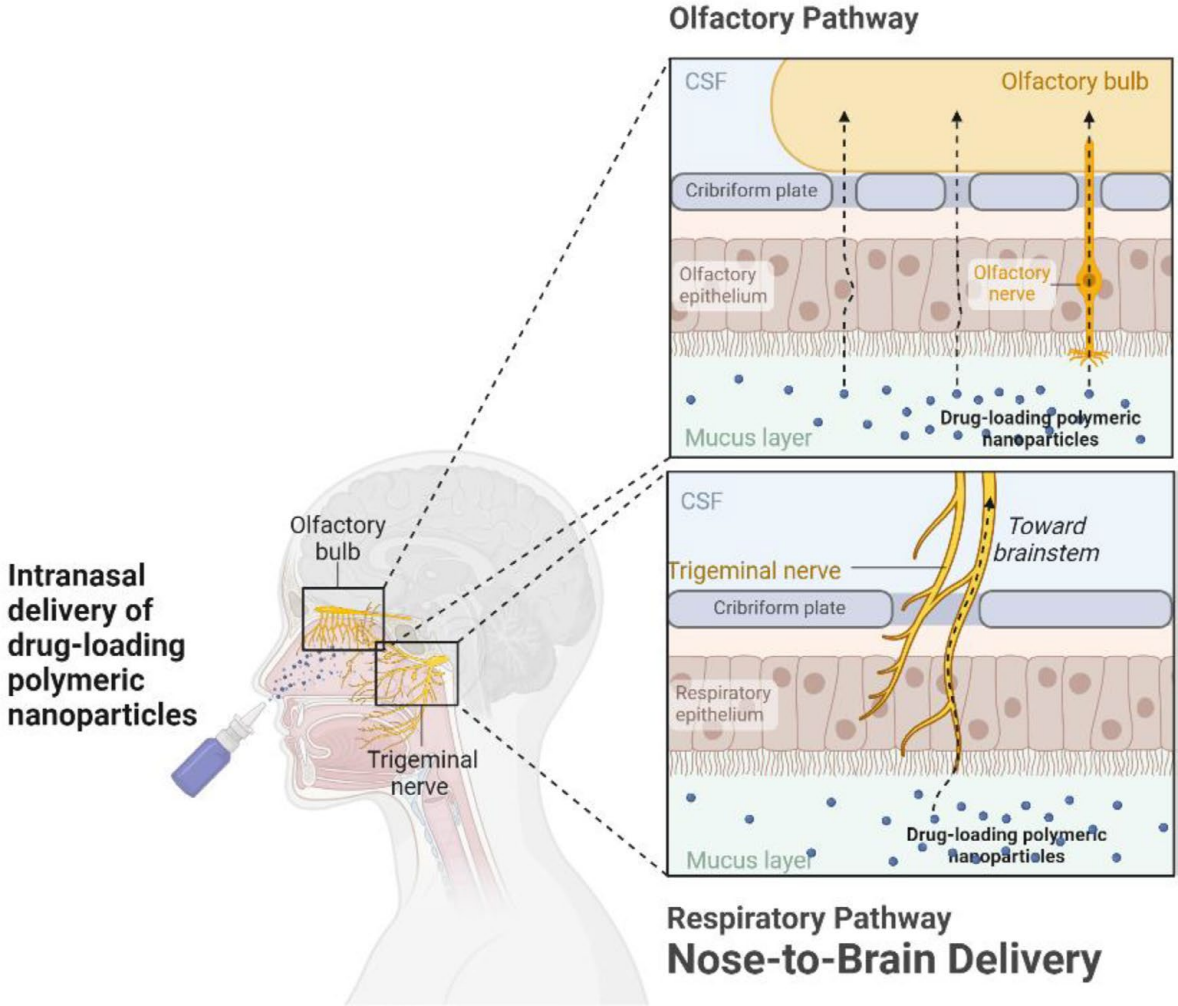
Schematic representation of the intranasal administration of polymeric nanoparticle‐loaded drugs to the brain. The nasal cavity consists of the vestibular region, the respiratory region and the olfactory region. The vestibular region is the outermost one, with the role of protecting the nasal cavity from harmful external agents. The respiratory region is highly vascularised and includes the trigeminal nerve. The olfactory nerve exists in the olfactory region and transports substances to brain stem. Nose to brain delivery of polymeric nanoparticle–loaded drugs is predominantly through the olfactory pathway and respiratory pathway.

In comparison with indirect administration routes, intracerebroventricular route directly injects medications to the brain. However, the strategy is invasive and not feasible for repeated drug administration [54]. Another direct and non‐invasive pathway as intranasal (nose to brain) administration has gained particular attention [55, 56]. This pathway involves the olfactory and trigeminal nerve systems that originate in the brain and end in the nasal cavity, namely, the olfactory neuroepithelium and the respiratory epithelium, respectively [57, 58]. The anatomy demonstrates that the nasal cavity, lined by the nasal mucosa, is the only contact region between the CNS and the external environment and therefore the most direct and non‐invasive way of accessing the brain [58, 59, 60]. With nose to brain route, the drug loaded in polymeric nanoparticles can be directly transported to the brain via olfactory pathway and respiratory pathway (Figure 3), which minimises its systemic exposure and dramatically elevates the utilisation rate of therapeutic agents. Furthermore, abundant vascularisation increases the drug absorption rate and allows a rapid onset of the therapeutic effect [55, 61]. In this context, polymeric nanoparticles present promising carriers for nose to brain drug delivery in neurodegenerative diseases. However, limitations still exist as the low dose of drug uptake by the brain as a consequence of the low permeability of medications through the mucosa, mucociliary clearance and enzymatic degradation [62]. Frequent mucosal stimulation is also an issue to take into consideration. Therefore, it is necessary to develop strategies for the drug delivery systems to improve drug absorption from the olfactory and respiratory regions of the nasal cavity to the brain. Specifically, a series of factors involved in the mechanism of administration need to be taken into account in the medication administration for the treatment of neurodegenerative disorders via the intranasal route [63]. Firstly, the size of the nanoparticles should be in a range that allows migration through the mucous membranes to the CNS. As reported, nanoparticles with an average size of up to 200 nm were efficiently transported transcellularly via the intranasal route [64]. Another study further limited the maximum usable diameter to 100 nm, especially via the olfactory axons to the brain [65, 66]. Another factor influencing this route of administration is the surface modification of the nanoparticles to facilitate drug delivery into the brain. PEG coating is the most commonly used modification to assist the adhesion of complexes to the nasal mucosa, and low‐molecular‐weight PEG contributes to nanoparticle movement [56]. Furthermore, surface modifications with ligands, especially cell‐penetrating peptides, have been shown to be effective for enhancing intranasal drug delivery. Gartziandia et al. [67] reported that coating with cell‐penetrating peptides such as Tat and penetratin dramatically elevated the nose to brain delivery efficiency in two types of polymeric nanoparticles. The extensively studied lecithins have been limited due to its extreme immunogenicity. To improve stability and transmembrane penetration and increase the residence time of the formulation in the nasal cavity, suitable ligands without obvious toxicity or immunogenicity are necessary for these polymeric nanoparticle–based systems [63, 68, 69].

## 3 Polymeric Nanoparticles as Drug Carriers in AD

**FIGURE 4:**
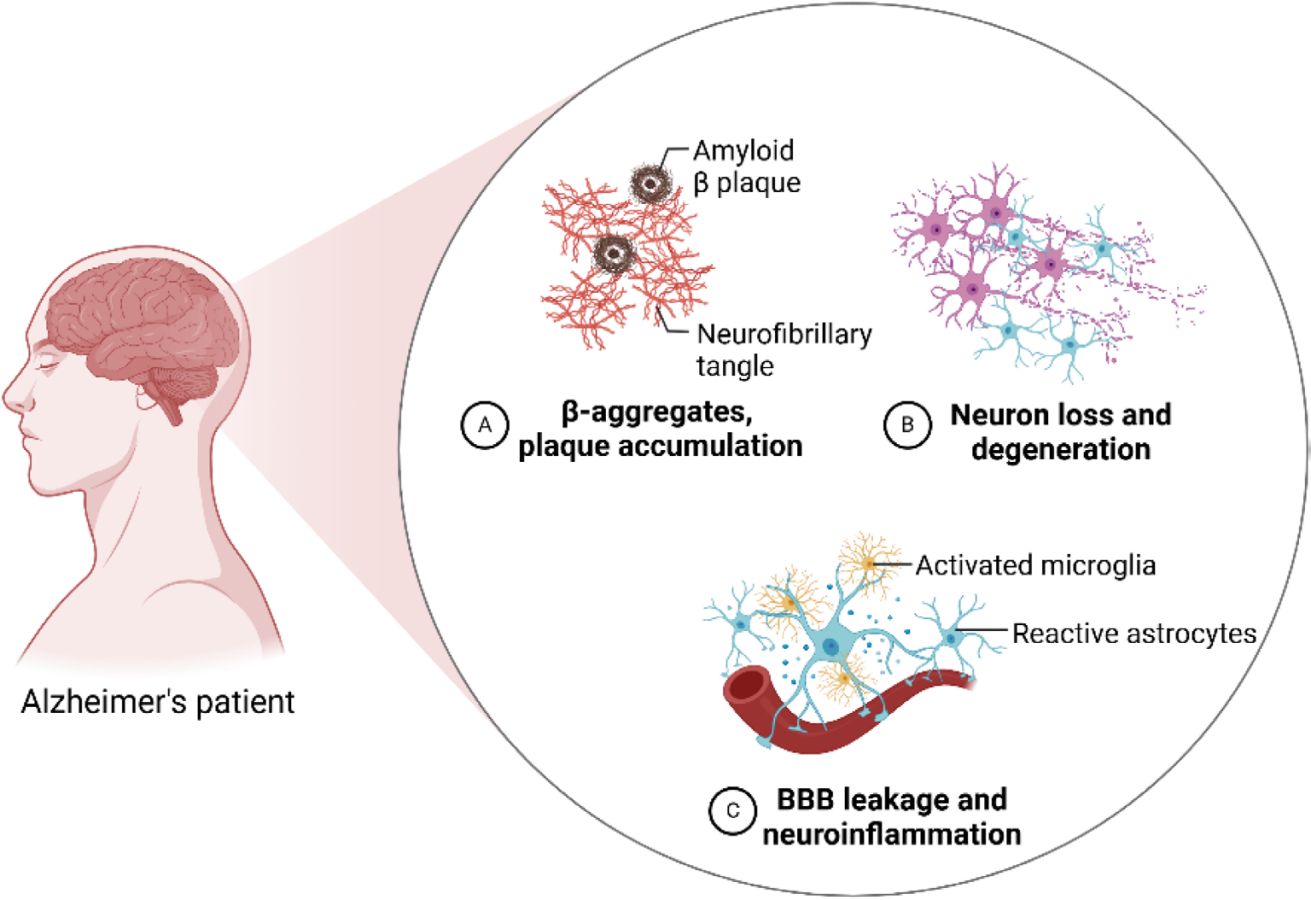
Neuropathological features of AD.

AD, as the main cause of dementia, is the most common neurodegenerative disease with the symptoms including progressive memory loss, cognitive impairment and pronounced decline of intellectual capacities [70]. A dementia prevalence of about 50 million people worldwide was estimated in 2018, which was projected to triple by 2050 [71]. The most recent data estimate that dementia prevalence in Europe will double by 2050 [70]. The aetiology of AD has not yet been fully elucidated although it is characterised by the deposition of amyloid‐beta (Aβ) protein and neurofibrillary tangles (NFTs) of tau proteins, along with neuron loss and degeneration (Figure 4). The underlying pathogenesis involves BBB leakage, increased inflammatory response, accumulation of reactive oxygen species (ROS), acetylcholine (ACh) deficiency, lack of neurotropic factors, genetic predisposition, etc. [72, 73, 74, 75] Accordingly, a wide variety of therapeutic agents have been developed for AD treatment, including cholinesterase and phosphodiesterase inhibitors, anti‐inflammatory drugs, antioxidants, tau hyperphosphorylation (e.g., GSK3 serine–threonine kinase inhibitors, such as thiazolidinediones), intracellular NFT inhibitors (e.g., methylene blue) and neurotrophins (e.g., brain‐derived neurotropic factor [BDNF]) [76, 77]. However, the development of these medications has been limited by the strictly selective nature of the BBB, which stops the majority of drugs from entering the brain [15]. Systemic administration of these drugs can cause severe peripheral side effects [78, 79], which also contribute to the limitation in the medications of AD. To overcome the obstacles, polymeric nanoparticles have come up as ideal drug carriers in AD because they possess markable advantages such as excellent BBB penetration ability and specific targeting through flexible surface modification or/and suitable administration route [80]. Notably, an innovative approach has been proposed as nose to brain delivery, which is capable of directly transporting medications to the brain without bypassing the BBB. In this scenario, a broad spectrum of research on polymeric nanoparticle–based drug delivery to the CNS via intranasal pathway in AD has been carried out [81]. As listed in Table 1, the widely investigated polymers include chitosan, poly(lactic‐co‐glycolic acid) (PLGA), polylactic acid (PLA), polyamidoamine (PAMAM) dendrimers, polycaprolactone (PCL) and poly(alkyl cyanoacrylate) (PACA) [96, 97, 98].

**TABLE 1:** Polymeric nanoparticles for drug delivery in preclinical studies of AD.

Drug	Nanoparticle material	Administration route	Models	Remarks	References
Galantamine hydrobromide	Chitosan	Intranasal	Wistar rats	Significantly decreased the acetylcholinesterase protein level and activity in the brains	Hanafy et al. [82]
Galantamine hydrobromide	Chitosan	Intranasal	Wistar rats	No influence on oxidative stress or neuroinflammation	Kandil et al. [83]
Galantamine	Thiolated chitosan	Intranasal	Amnesia mice	Significant recovery in pharmacodynamic performances and acetylcholinesterase activity	Sunena et al. [84]
Resveratrol	Chitosan	—	Obesity‐related AD mice	Improved glucose homeostasis, oxidative stress, gut microbiota and neuroinflammation	Yang et al. [85]
PLGA	PLGA	Intracerebroventricular	Mouse primary cortical neurons, 5xFAD mice	Suppressed spontaneous aggregation and triggered disassembly of preformed Aβ, attenuated memory deficits	Anand et al. [86]
Fucoxanthin	PEG–PLGA	Intravenous	Aβ oligomer‐induced AD mice	Inhibited the formation of Aβ fibrils and oligomers, prevented cognitive impairments	Yang et al. [87]
Berberine	Tet‐1 peptide‐PLGA	Intravenous	Sporadic AD rats	Attenuated hippocampal damage, enhanced cognitive performance, reduced Aβ42, Tau phosphorylation, and proinflammatory responses, restored neuroplasticity, cholinergic and monoaminergic function	Saleh et al. [88]
Cannabidiol and brain‐derived neurotrophic factor	Chitosan‐coated PLGA	—	Primary astrocytes, primary neurons	Excellent performances in the encapsulating ability, sustained release and transfection efficiency	Mahanta et al. [89]
α‐Mangostin	PEG–PLA	Intravenous	SAMP8 and APP/PS1 transgenic mice	Improved drug biodistribution in both the brain and liver, enhanced brain clearance of Aβ1‐42, reduced Aβ deposition, attenuated neuroinflammatory responses, ameliorated neurologic changes, reversed behavioural deficits	Yao et al. [90]
H102 and NAP peptides	PEG–PLA	Intravenous	3 × Tg‐AD transgenic mice	Prevented neuroinflammation, reversed the neuronal damage, restored cognitive performance	Guo et al. [91]
Rifampicin	PEG–PLA	Intravenous	APP/PS1 mice	Reduced Aβ deposition in hippocampal and cortex, improved the damage of synaptic ultrastructure, increased the expression of PSD95 and SYP, reduced necrosis of neurons	Zhou et al. [92]
Tacrine	PAMAM dendrimers	—	Human red blood cells, neuro‐2a cell line, zebrafish larvae	Reduced amyloid plaque formation and aged microglia surrounding the plaque, reversed learning deterioration and spatial memory deficits	Igartua et al. [93]
Curcumin	Polycaprolactone	Intravenous	AD mice	Enhanced spatial learning and memory abilities, increased the dendritic segment densities, restrained the activation of microglia and astrocytes, reversed cognitive function	Gu et al. [94]
Rivastigmine	Polycaprolactone	Intravenous	Rats	Increased brain uptake, relieved memory loss	Mohamadpour et al. [95]

**FIGURE 5:**
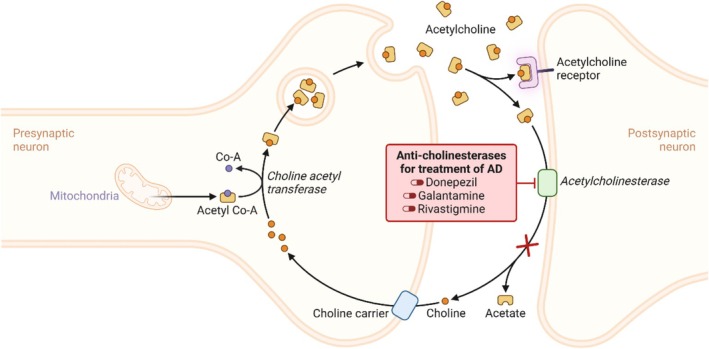
Anti‐cholinesterase mechanism of action in AD.

Among polymeric nanocarriers, chitosan nanoparticles emerge as biodegradable and stable vectors for the delivery of CNS medications. Considering their mucoadhesive character and intrinsic bioactivity, chitosan nanoparticles can not only increase permeability through the mucosa and reduce mucociliary clearance to promote drug delivery efficiency via the intranasal route but also act as anti‐Alzheimer therapeutics themselves [76]. To give examples, when the rats were subjected to intranasal administration of galantamine hydrobromide (GH)/chitosan complex nanoparticles, particles were found in neurons and no toxicity was detected. Compared with oral and nasal GH solutions, a significant decrease of acetylcholinesterase protein level and activity was identified in the brains of the nanoparticle group [82]. A similar method was used in another study, which reported that neither oxidative stress nor neuroinflammation was found in rats exposed to intranasal administration of GH/chitosan complex nanoparticles [83]. In another study, AD mice showed significant recovery in pharmacodynamic performances and acetylcholinesterase activity after exposing to the intranasal delivery of galantamine‐loaded thiolated chitosan nanoparticles [84]. GH is one of the drugs used for the treatment of mild to moderate AD approved by the Food and Drug Administration (FDA). It is a reversible competitive inhibitor of cholinesterase, the enzyme responsible for inactivating the neurotransmitter acetylcholine [99, 100]. It can improve AD conditions through repressing the inactivation of cholinesterase in the brain (Figure 5). Other ways to improve AD symptoms have been reported. A brain‐targeted peptide was conjugated to the surface of resveratrol‐loaded chitosan nanoparticles to facilitate targeted delivery in obesity‐related AD mice. The results indicated that peptide modification substantially improved glucose homeostasis, oxidative stress and neuroinflammation in the brain [85]. Resveratrol is a polyphenol with antioxidant or anti‐inflammatory properties in age‐related diseases [101]. Brain‐targeted peptide conjugation contributes to targeted delivery to the CNS.

PLGA is another widely investigated polymer for the drug delivery in AD. In addition to good biocompatibility and biodegradability, it is able to increase the stability of the transported payload and allow the encapsulation of the drugs to be later released. PLGA nanoparticles were found to be able to not only suppress the spontaneous aggregation but also trigger the disassembly of preformed Aβ aggregates in both in vitro cell models and in vivo animal models, which finally was performed as attenuated memory deficits [86]. PLGA nanoparticles coated with PEG represent better properties as drug delivery carriers in AD. For instance, fucoxanthin‐loaded PLGA–PEG nanoparticles demonstrated favourable in vitro and in vivo properties in AD. An obvious nanoparticle uptake and decreased Aβ oligomer‐induced neurotoxicity in neurons and microglia were observed, and ameliorated cognitive performance was verified in AD mice [87]. Compared with free berberine, berberine‐loaded PLGA/Tet‐1 peptide nanoparticles showed better bioavailability, absorption and brain uptake and restored neuroplasticity, cholinergic and monoaminergic functions in a rat model of sporadic AD [88]. Chitosan‐coated PLGA nanoparticles conjugated with mannose, which can specifically target the glucose transporter‐1 receptor abundantly present in the BBB, were adopted for the codelivery of cannabidiol and BDNF to the brain. Excellent performances in the encapsulating ability, sustained release and transfection efficiency were observed [89]. Fucoxanthin, a marine carotenoid, possesses neuroprotective, antioxidant and anti‐inflammatory properties and able to reduce amyloid‐β production and tau hyperphosphorylation [102]. Berberine, an important protoberberine alkaloid widely used in traditional Chinese medicine for hundreds of years, exhibits a neuroprotective role in AD by inhibiting oxidative stress and neuroinflammation [103, 104]. Cannabidiol, the major non‐psychoactive component of cannabis, has attracted attention due to its antioxidant and anti‐inflammatory properties and the ability to reduce the accumulation of Aβ and hyperphosphorylation of tau in AD [105, 106]. BDNF is a well‐known growth factor with a vital role in facilitating nerve growth and maturation and regulating synaptic transmission and plasticity in human brain. Mounting evidence has identified that BDNF depletion is associated with tau phosphorylation, Aβ accumulation, neuroinflammation and neuronal apoptosis in AD [107, 108, 109]. PLGA–PEG nanoparticles facilitated the brain delivery of these medications in AD, probably as PEG coating stabilised the particles before they arrived at the brain. Tet‐1, a 12‐amino acid peptide, can interact specifically with neurons, which may help improve the drug delivery efficacy.

PLA is also a widely studied polymer for the preparation of nanoparticles to deliver drugs to the CNS, especially through the nose to brain pathway. PEG–PLA nanoparticles attracted much attention as carriers due to its suitable safety and clinical translation ability [110, 111]. The administration of α‐mangostin‐loaded PEG–PLA nanoparticles improved the drug biodistribution in both the brain and liver, enhanced the brain clearance of Aβ (1–42), reduced Aβ deposition, attenuated neuroinflammatory responses, ameliorated neurologic changes and reversed behavioural deficits in AD model mice [90]. In another study, PEG–PLA nanoparticles were adopted for the delivery of combined peptide drugs to the brain [91]. They generated synergistic therapeutic effects on Aβ, p‐tau and their linkage and effectually prevented neuroinflammation, reversed the neuronal damage and restored cognitive performance in 3 × Tg‐AD transgenic mice. Moreover, PLA–PEG‐Gd/Mal‐RVG29 nanoparticles were formulated for the loading of rifampicin [92]. The administration of drug‐loaded nanoparticles reduced Aβ deposition in hippocampal and cortex of APP/PS1 mice, improved the damage of synaptic ultrastructure, increased the expression level of PSD95 and SYP, as well as reduced the necrosis of neurons [92]. Rifampicin is a type of neuroprotective antibiotic that may simultaneously modulate the neuroinflammatory response and Aß metabolism in AD. PEG–PLA nanoparticle encapsulation improved its stability in the circulation, and targeting ligand modification contributed to its penetration across the BBB.

The application of other polymers as drug delivery vectors has also been explored. Tacrine, a licensed drug for the treatment of mild and moderate AD, reduced the cytotoxicity of tacrine on the Neuro‐2a cell line when co‐administrated with PAMAM and in vivo co‐administration reduced the morphological and hepatotoxic effects of tacrine in zebrafish larvae [93]. PCL nanoparticles coated with red blood cell membrane were synthesised and conjugated with a functional peptide for the brain delivery of curcumin, a therapeutic agent for AD [94]. The administration induced a series of positive effects including enhanced spatial learning and memory abilities, increased the dendritic segment densities, restrained the activation of microglia and astrocytes and reversed cognitive deficits [94]. Another study reported that PCL‐based nanoparticles delivered rivastigmine to rat brains and triggered the increased brain uptake and relieved memory dysfunction [95]. Similarly, PEGylated PACA nanocarriers were synthesised and functionalised with ligand and exhibited higher affinity toward Aβ (1–42) species and inhibited its aggregation [112]. Collectively, a large number of efforts have been made on the treatment strategy of AD, with the majority of them focused on acetylcholinesterase inhibition and Aβ clearance strategy. However, these strategies are all symptomatic, compared with which prevention and early intervention at the genetic level prior to the clinical symptoms are of greater significance.

As we know more and more about the roles of nucleic acids including DNA, message RNA, microRNAs (miRNAs) and small interfere RNA (siRNA) [113, 114], nucleic acid drugs have been widely investigated for gene therapy in AD, especially with polymeric nanocarriers [115, 116]. The cellular phase is considered as the preclinical phase of AD [117, 118]. Alterations in neurons, microglia and astrocytes drive the insidious progression of the disease before cognitive impairment is observed [119]. It was found that the p16^ink4a^ expression was increased in microglia near amyloid plaques in brain tissues from patients with AD and AD mice [120]. p16^ink4a^ siRNA delivered by PLGA nanoparticles reduced amyloid plaque formation and the number of aged microglia surrounding the plaque and reversed learning deterioration and spatial memory deficits [120]. Park et al. [121] prepared nanocomplexes composed of the R7L10 peptide complexed with Cas9‐sgRNA ribonucleoprotein targeting the BACE1 gene. The intrahippocampal injection of the nanocomplexes lowered Aβ levels and ameliorated memory impairment in *5xFAD* and *App* knock‐in AD transgenic mice. In a study by Li et al. [122], the lesion‐recognising nanoparticles consisted of rabies virus glycoprotein peptide–modified mesenchymal stem cell–derived exosomes as the shell and a ROS‐responsive polymer loaded with siRNAs as the core. They exerted a positive effect on regulating the phase of neurons and astrocytes, which resulted in better restoration of memory deficits in 3 × Tg‐AD mice. A D3‐peptide‐conjugated nanopolymer was reported to be able to achieve the neuron‐selective delivery of miRNA and serve as an efficient brain delivery vehicle in AD mouse models [123]. In summary, polymeric nanoparticle–based gene delivery strategies possess great potential to modify AD development at the genetic level.

## 4 Polymeric Nanoparticles as Drug Carriers in PD

PD is the second‐most common neurodegenerative disease with a global prevalence of more than six million individuals. The prevalence has been predicted to double over the next generation, which makes PD one of the leading causes of neurological disability [124, 125]. The pathological hallmark of PD consists of neuronal loss and the formation of α‐synuclein‐containing proteinaceous aggregates in neurons of the substantia nigra, known as Lewy bodies and Lewy neurites [126, 127]. However, the pathogenesis of PD remains elusive despite plenty of efforts been made [128, 129]. Current pharmacological strategies for PD are listed in Table 2, with a focus on replenishing dopamine levels in the depleted striatum, which provides a remarkable relief from motor and some non‐motor symptoms [130]. These medications include levodopa (L‐dopa), dopamine receptor agonists (e.g., pramipexole, rotigotine) and inhibitors of dopamine catabolic enzymes (e.g., selegiline, rasagiline) [131]. However, these treatments are limited because of several drawbacks such as instability of the drugs, difficulty in crossing the gut and BBB, dispersal of dopamine to unexpected regions and the non‐physiological stimulation of dopamine receptors [132, 133]. Polymeric nanoparticles for drug delivery have been designed to tackle these problems.

**TABLE 2:** Pharmacological strategies for PD treatment.

Treatment strategy	Therapeutic agent
Dopaminergic therapy	Levodopa, e.g., L‐dopa, dopamine
	Sopamine receptor agonists, e.g., bromocriptine, pramipexole, ropinirole, rotigotine
	Inhibitors of dopamine catabolic enzymes, e.g., selegiline, rasagiline
Nerve growth factors	Nerve growth factor, GDNF and VEGF
Gene delivery systems	siRNA complex, hGDNF gene, miR‐124
Repurposing natural products	Curcumin, levodopa, nicotine, resveratrol

Chitosan, the deacetylated form of chitin, is ideal for the preparation of polymeric nanoparticles due to its biocompatibility, biodegradability, low toxicity and mucoadhesive nature [134]. Trapani et al. [135] utilised chitosan nanoparticles to deliver dopamine in a rat model. The intraperitoneal administration of dopamine‐loaded chitosan nanoparticles resulted in a dose‐dependent increase in dopamine release in the striatum. Intranasal administration of chitosan nanoparticles encapsulating pramipexole dihydrochloride was reported to display improved motor deficits in behavioural tests, increased antioxidant activity and elevated dopamine levels in the brain [136]. Rotigotine‐loaded chitosan nanoparticles exerted positive effects on both in vitro cells and in vivo animal model of PD, which performed as improved enzyme activities and alleviated motor deficits [137]. Similarly, the intranasal administration of selegiline‐loaded chitosan nanoparticles induced improved motor performance and increased brain dopamine [138].

Synthetic polymers such as PLGA, PCL, PACA and PLA have also been used in the drug delivery to the brain [96, 97, 98]. PLGA can encapsulate and protect both water‐soluble and lipid‐soluble drugs and release drugs via a combination of passive diffusion and gradual matrix degradation [139]. Dopamine was entrapped in PLGA nanoparticles and intravenously administrated to a 6‐hydroxydopamine rat model, which induced elevated brain dopamine and reduced neuronal death [140, 141]. To further improve the performances of synthetic polymer–based nanoparticles, they have been conjugated with various ligands or substances to achieve different objectives. PEG modification is widely known to improve the properties of nanoparticles by forming a hydrophilic protective layer on the surface, thus decreasing the clearance of nanoparticles from the circulation by the immune system [142]. PEG–PCL nanoparticles loaded with ginkgolide B were formulated, and they increased brain uptake and improved the drug releasing profile [143]. Wheat germ agglutinin is a plant lectin capable of strongly binding to glycosylated cell membranes [144]. Conjugation of wheat germ agglutinin to PLGA nanoparticles increased brain drug delivery and prolonged L‐dopa release time by improving absorption and retention through the nasal cavity [145]. Albumin, as the most abundant protein in plasma, can help nanoparticles to be ‘accepted’ by the circulation of human body. Combining albumin with PLGA nanoparticles improved the stability and half‐life time of dopamine, thereby restoring the dopamine level in the striatum in a mouse model of PD [146]. Lactoferrin can bind to not only negatively charged cell membranes with its positive charges but also specific lactoferrin receptors overexpressed in the substantia nigra, striatal dopaminergic neurons and BBB endothelial cells, thereby facilitating the targeted transcytosis of nanoparticles [147, 148]. In a study, PLGA–PEG nanoparticles surface‐modified with lactoferrin were used for rotigotine delivery in vitro and in vivo. Better uptake was found in 16HBE and SH‐SY5Y cells, and increased brain delivery of rotigotine was observed in the striatum of mice [149]. Another similar study reported PEG–PLGA nanoparticles coated with borneol and lactoferrin for the targeted intranasal administration of dopamine. The modification facilitated the endocytosis of nanoparticles in 16HBE cells. And brain dopamine levels were elevated and striatum damage were significantly alleviated in a PD model of rats [150]. However, these attempts are all used for the symptom treatment of PD, which is not able to resolve the irreversible cognitive impairment. Therefore, we are seeking methods for the prevention or early interference of PD.

**FIGURE 6:**
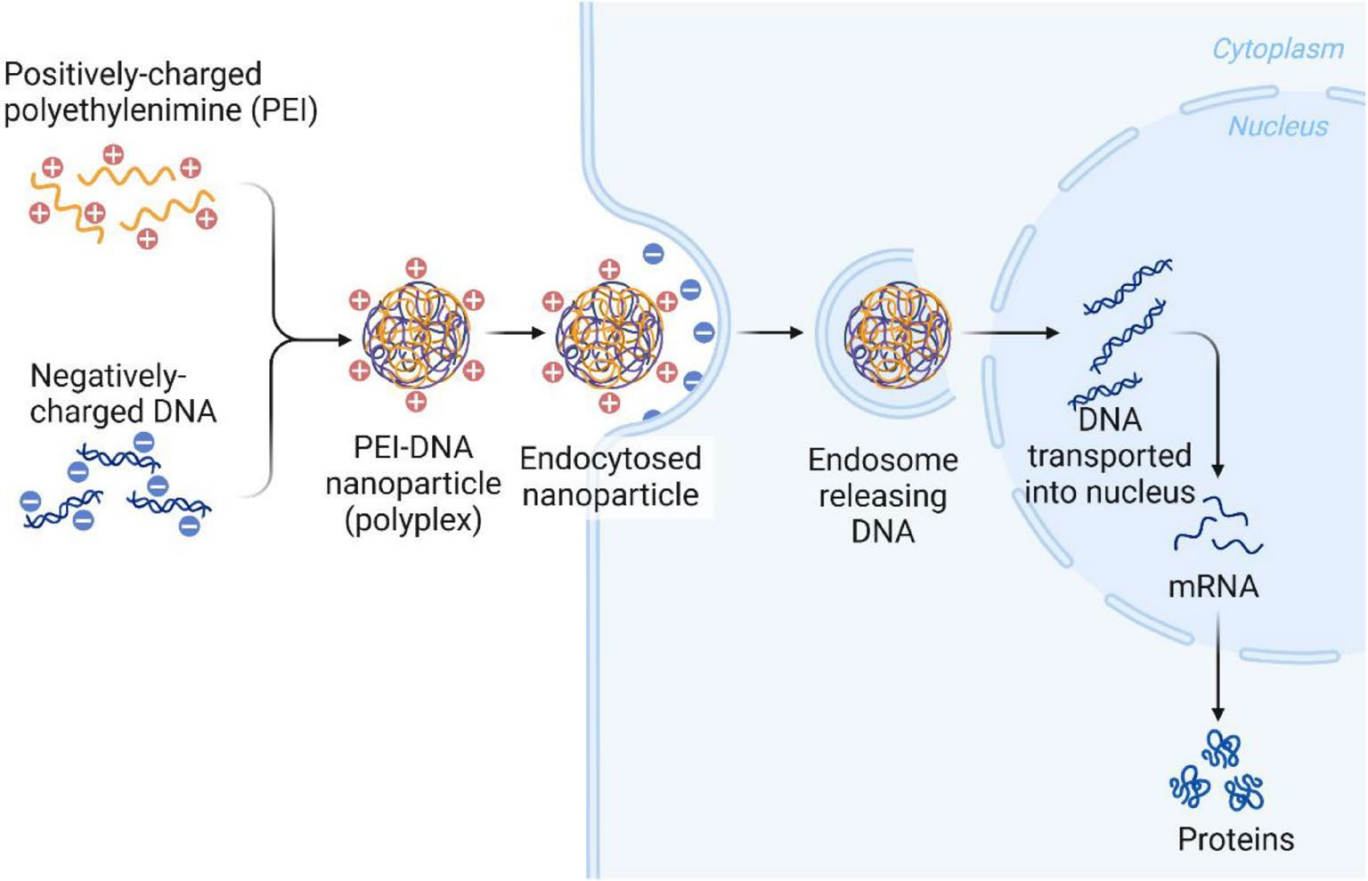
Schematic representation of PEI‐mediated gene delivery.

As the gene editing techniques develop, gene therapy is applied in the investigation of PD pathogenesis, especially polymeric nanoparticle–based gene delivery. Polyethyleneimine (PEI) is a common cationic polymer used in gene delivery (Figure 6). It was reported that PEI successfully incorporated siRNA targeting α‐synuclein. Intracerebroventricular infusion of the PEI/siRNA complex resulted in a substantial reduction of α‐synuclein levels in the striatum of mice [151]. In comparison with PEI, PAMAM has the advantages of less cytotoxicity and better transfection efficiency [152]. PAMAM nanoparticles modified with lactoferrin and PEG delivered human GDNF gene in a rotenone‐induced rat model of PD, which led to better locomotor activity, decreased dopaminergic neuronal expression and increased dopamine levels [153]. In another study, miR‐124 was incorporated into a PLGA‐based nanocarrier and its uptake by stem cells was improved, which was supported by the induction of stem cell differentiation and the neuroprotective effects in 6‐OHDA‐induced PD mice [154]. Compared with traditional drug treatment, gene therapy stands out as it is a strategy to interfere the disease development from the genetic level, which happens prior to the presence of clinical symptoms.

## 5 Polymeric Nanoparticles as Drug Carriers in Other Neurodegenerative Diseases

HD is the most frequent autosomal‐dominant neurodegenerative disorder. The proposed pathogenesis of HD includes toxicity from full‐length expanded huntingtin and N‐terminal fragments of huntingtin, which are both prone to misfolding due to proteolysis, aberrant intron‐1 splicing of the HTT gene and somatic expansion of the CAG repeat in the HTT gene [155]. Therapies targeting huntingtin DNA and RNA, clearance of huntingtin protein, DNA repair pathways, anti‐inflammation methods and stem cell transplantation are all listed as potential strategies for HD treatment [156]. However, there are not many studies adopting polymeric nanoparticles. Chitosan‐based nanoparticles loaded with anti‐HTT siRNA was intranasally administrated to a transgenic YAC128 mouse model of HD, which resulted in a decrease of HTT mRNA expression at over 50% [157]. Primary hippocampal cells were incubated with hybrid nanoparticles composed of cholesterol and PLGA modified with g7 ligand. This induced the increased uptake and modified expression of synaptic receptors that could be beneficial in HD [158]. Thus, there are still much space for the development of polymeric nanoparticle–based drug delivery, especially gene delivery in HD. Edaravone is a potent antioxidant drug approved for ALS treatment, which is limited by its short biological half‐life and poor water solubility. Lu et al. [159] prepared edaravone‐loaded PLGA nanoparticles, and the intranasal administration showed higher and more sustained brain uptake compared with intravenous administration. 2‐(Phosphonomethyl)pentanedioic acid, a potent glutamate carboxypeptidase II inhibitor that can robustly block glutamate release from *N*‐acetyl‐aspartyl‐glutamate, was attached to a hydroxyl PAMAM dendrimer delivery system [160]. The formulation delayed muscle function loss and denervation in a mouse model of ALS [160]. Another study reported that avidin–nucleic‐acid‐nanoassembly improved the drug passage through the BBB in a murine model of ALS [161]. Astragaloside IV has anti‐inflammatory, antioxidant, remyelination and neuroprotective activities in MS, while it is restricted by its poor permeability, relatively high molecular weight and low lipophilicity. Zhao et al. [162] formulated β‐asarone‐modified astragaloside IV‐loaded chitosan nanoparticles. They significantly alleviated behavioural performances, suppressed inflammatory infiltration and astrocyte/microglial activation, reduced demyelination and increased remyelination in EAE mice, a typical animal model of MS. In another mouse model of MS, myelin antigens delivered by PLGA nanoparticles induced robust tolerance and long‐term comprehensive disease protection [163]. Taken together, there are not enough investigations on the application of polymeric nanoparticles in HD, ALS and MS, although their advantages and potential have been highlighted.

## 6 Current Research Challenges and Future Perspectives

Polymeric nanoparticles illustrate significant efficacy in various preclinical studies, including in vitro, ex vivo and animal models of neurodegenerative diseases, especially AD and PD. Considering the different features of intravenous and intranasal administration [62], appropriated approaches should be employed to bring them closer to clinical applications. For intravenous administration, flexible ligand modifications, decreased peripheral side effects and enhanced BBB penetration are the preferred properties. Nevertheless, there are big challenges in the suitable modifications to achieve eligible goals in both the stability in the circulation and the escape ability from the immune system. For the nose‐to‐brain route, the direct transport without bypassing the circulation stands out. However, the limited dose of drug uptake and frequent mucosa stimulation are issues to resolve. Enzymatic inhibitors and absorption promoters (e.g., cell‐penetrating peptides) can be good choices as modification ligands. Therefore, both the administration routes and the polymeric nanoparticle vectors are still under investigations with a large number of preclinical studies. Numerous challenges exist on the way to clinical application. From another perspective, the current medications for neurodegenerative disorders are only for symptomatic treatment and prevention or early intervention is lacking, necessitating a therapeutic strategy to interfere diseases prior to the occurrence of irreversible neurological damage. Nucleic acid–based drugs (gene therapy) can modify the diseases from the genetic level, which will then stop the appearance of the following clinical symptoms [164]. However, translating this method to human effectiveness is not straightforward due to the complexity, safety and variability of gene delivery within the human body, particularly in diseased states [165]. Numerous efforts are required to make to bring the strategy to the clinical application. In this context, better understanding of the disease pathology and the in‐depth exploration of carriers for therapeutic agents including traditional drugs and nucleic acid drugs will definitely contribute to the treatment and even the early intervention of neurodegenerative diseases.

## Author Contributions

Conceptualisation, preparation and revision of the manuscript, L.J., L.N., Y.D., N.H.; methodology, L.J., L.N.; manuscript preparation, L.J., Y.D. All authors have read and agreed to the published version of the manuscript.

## Conflicts of Interest

The authors declare no conflicts of interest.

## Data Availability

The authors have nothing to report.
